# The International Diabetes Federation World Diabetes Congress 2015

**DOI:** 10.17925/EE.2015.11.02.66

**Published:** 2015-08-19

**Authors:** Bernard Zinman

**Affiliations:** Programme Committee Chair of the World Diabetes Congress 2015

## Abstract

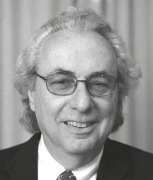

**Bernard Zinman**, Programme Committee Chair of the International Diabetes Federation (IDF) World Diabetes Congress 2015, talks about the scientific programme highlights, the experience of attending the Congress and his involvement in diabetes care and research. Bernard Zinman is Director of the Leadership Sinai Centre for Diabetes and holds the Sam and Judy Pencer Family Chair in Diabetes Research at Mount Sinai Hospital and the University of Toronto, Canada. He is Professor of Medicine at the University of Toronto and Senior Scientist at the Samuel Lunenfeld Tanenbaum Research Institute, Mount Sinai Hospital, Ontario, Canada.

## Q&A with Bernard Zinman, Chair, International Diabetes Federation World Diabetes Congress 2015 Programme Committee

### 1. What are going to be the highlights of the congress scientific programme?

The International Diabetes Federation (IDF) World Diabetes Congress (WDC) 2015 will be held in an outstanding venue in Vancouver, Canada. The programme will comprise symposia, debates, workshops, meet-the-expert and poster discussion sessions, and will present recent advances in six distinct streams including: Basic and Clinical Science, Diabetes in Indigenous Peoples, Education and Integrated Care, Global Challenges in Health, Public Health and Epidemiology and Living with Diabetes.

### 2. Who should attend the congress and what can delegates expect from the experience?

Members of broad-based multi-disciplinary diabetes health care teams, individuals active in diabetes organisations, people with diabetes, diabetes-related industries and health ministries of various government sectors will all benefit from the diverse programme.

### 3. What makes the IDF WDC unique?

The uniqueness of the IDF WDC derives from its international flavour, the diverse background of its participants and the integration of cutting-edge research with education and clinical care. It truly is a unique event for people involved with every component of the diabetes community to meet and exchange ideas.

### 4. What was your motivation for becoming involved in diabetes care and research?

My background in biochemistry and passion for research directed me to a career as a clinician scientist in endocrinology with a major focus in diabetes.

**Figure:**
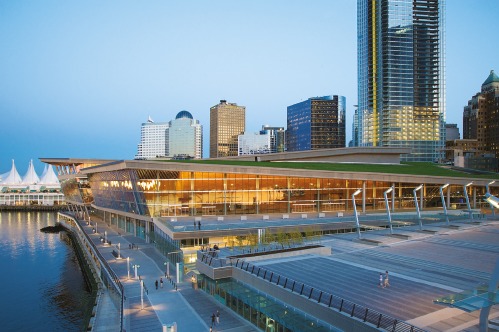
Vancouver Convention Centre East View.

### 5. Diabetes rates in North America are on the rise, what must we do to combat this?

To effectively address the epidemic of diabetes we need three approaches. First, we need to develop effective strategies to prevent diabetes with a focus on reversing the increasing rates of obesity and physical inactivity particularly as it relates to our children. Second, we need to develop and implement effective diabetes treatment regimens very early on in the course of the disease to prevent the devastating complications of diabetes. And finally, for those with diabetes and complications, we need to screen efficiently for these complications and apply effective treatment before they become disabling and affect the long-term outcomes and quality of life.

